# Trapping of normal EB1 ligands in aggresomes formed by an EB1 deletion mutant

**DOI:** 10.1186/1471-2121-6-17

**Published:** 2005-04-06

**Authors:** Nick P Riess, Kelly Milward, Tracy Lee, Matthew Adams, Jon M Askham, Ewan E Morrison

**Affiliations:** 1 CRUK Clinical Centre at Leeds, Division of Cancer Medicine Research, St James's University Hospital, Leeds LS9 7TF, UK; 2 Molecular Medicine Unit, University of Leeds, Clinical Sciences Building, St. James's University Hospital, Leeds LS9 7TF, UK

## Abstract

**Background:**

EB1 is a microtubule tip-associated protein that interacts with the APC tumour suppressor protein and the p150glued subunit of dynactin. We previously reported that an EB1 deletion mutant that retains both of these interactions but does not directly associate with microtubules (EB1-ΔN2-GFP) spontaneously formed perinuclear aggregates when expressed in COS-7 cells.

**Results:**

In the present study live imaging indicated that EB1-ΔN2-GFP aggregates underwent dynamic microtubule-dependent changes in morphology and appeared to be internally cohesive. EB1-ΔN2-GFP aggregates were phase-dense structures that displayed microtubule-dependent accumulation around the centrosome, were immunoreactive for both the 20s subunit of the proteasome and ubiquitin, and induced the collapse of the vimentin cytoskeleton. Fractionation studies revealed that a proportion of EB1-ΔN2-GFP was detergent-insoluble and ubiquitylated, indicating that EB1-ΔN2-GFP aggregates are aggresomes. Immunostaining also revealed that APC and p150glued were present in EB1-ΔN2-GFP aggregates, whereas EB3 was not. Furthermore, evidence for p150glued degradation was found in the insoluble fraction of EB1-ΔN2-GFP transfected cultures.

**Conclusion:**

Our data indicate that aggresomes can be internally cohesive and may not represent a simple "aggregate of aggregates" assembled around the centrosome. Our observations also indicate that a partially misfolded protein may retain the ability to interact with its normal physiological ligands, leading to their co-assembly into aggresomes. This supports the idea that the trapping and degradation of co-aggregated proteins might contribute to human pathologies characterised by aggresome formation.

## Background

EB1 is the prototypical member of a highly conserved family of proteins that localise to centrosomes and growing microtubule tips [1-3]. EB1 has been shown to directly interact with microtubules, the adenomatous polyposis coli (APC) tumour suppressor protein and the p150glued subunit of the dynein/dynactin microtubule motor complex [3-9]. In a previous study of a series of EB1 deletion mutants we noted that an EB1 protein lacking its *N*-terminal 100aa and fused at its *C*-terminus to GFP (EB1-ΔN2-GFP) spontaneously formed perinuclear aggregates in transfected COS-7 cells fixed and examined by immunostaining [3]. The present study represents a further characterisation of these aggregates.

This phenomenon was considered worthy of investigation for a number of reasons. Removal of the *N*-terminal 100aa of EB1 to generate EB1-ΔN2-GFP neatly removes one of the major structural features of the protein, a calponin homology (CH) domain implicated in the microtubule-binding ability of EB1 [3,9-11], while leaving the region that mediates the interactions with APC and p150glued intact. Consistent with this, EB1-ΔN2-GFP does not localise to microtubules in transfected cells whereas a GST-EB1-ΔN2 recombinant fusion protein binds to both APC and p150glued *in vitro *[3]. The behaviour of EB1-ΔN2-GFP in transfected cells might therefore reveal new information about EB1 folding and function. In addition, EB1-ΔN2-GFP aggregation resembled a cellular response to the presence of misfolded, proteolytically resistant proteins termed aggresome formation [see ref [12] for a recent review]. Aggresomes assemble around centrosomes in a process that requires microtubules and dynein/dynactin-mediated transport [13-15]. As EB1-ΔN2-GFP retains the ability to directly interact with p150glued, an examination of the aggregation of this protein in transfected cells might yield further insight into the process of aggresome formation. Furthermore, it seemed possible that the perinuclear EB1-ΔN2-GFP aggregates might represent a structure unrelated to aggresomes and instead arise from dominant-negative effects on the normal EB1/p150glued interaction. We therefore reasoned that detailed examination of EB1-ΔN2-GFP aggregation might shed further light on the normal function of this interaction within cells.

## Results

### EB1-ΔN2-GFP aggregate dynamics in living cells

**Figure 1:**
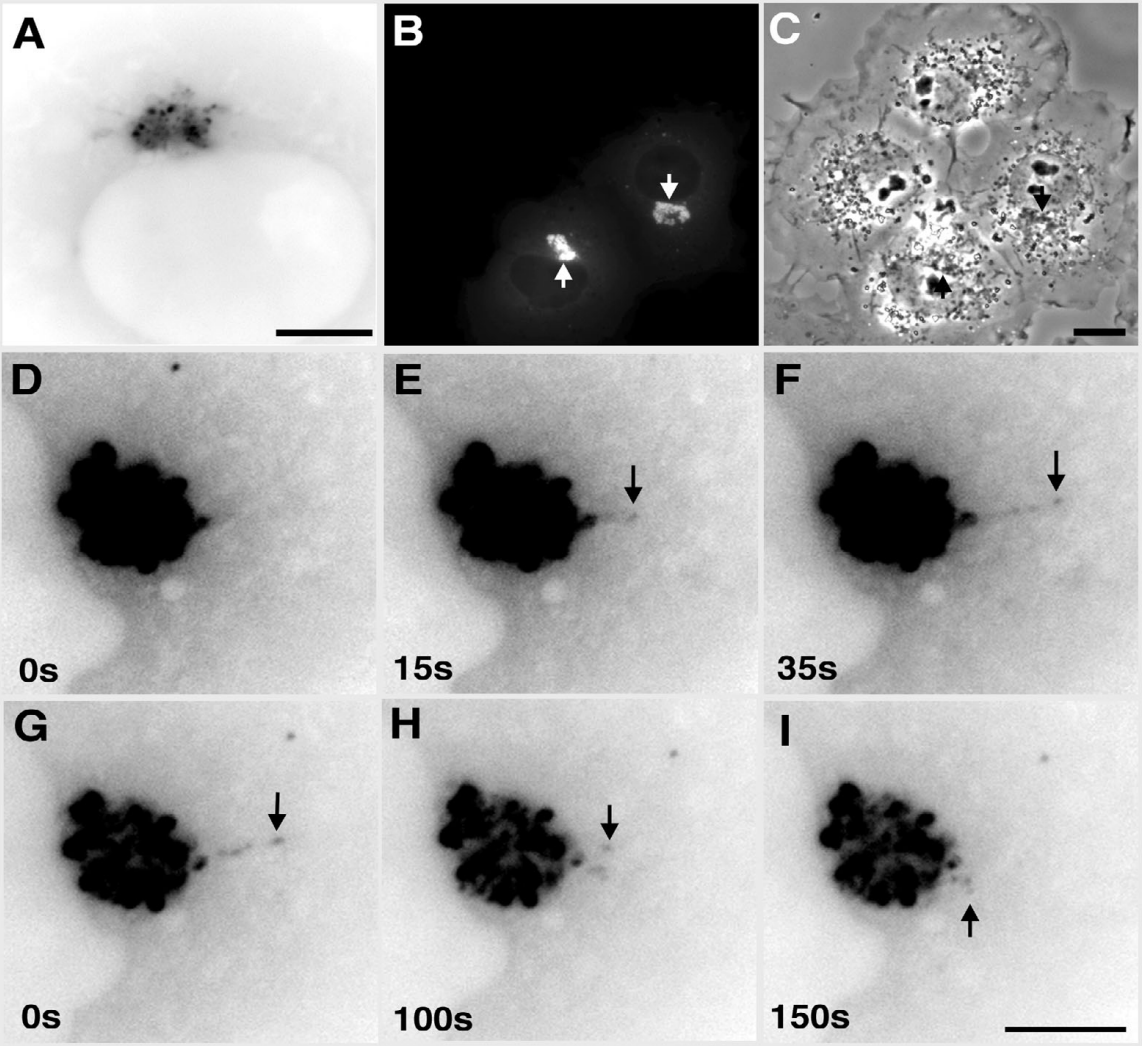
Live imaging of EB1-ΔN2-GFP aggregates in transfected COS-7 cells. Panel A. Single frame from additional file 1 showing the structure of EB1-ΔN2-GFP aggregates. Bar = 5 μm. Panel B. Single frame from additional file 2, sequence 1 showing aggresome (arrows) behaviour in two transfected cells. Panel C. Single frame from additional file 2, sequence 2 that shows the cells from sequence 1 imaged using phase-contrast microscopy to reveal adjacent untransfected cells. This sequence was begun 30s after the completion of sequence 1. Arrows indicate aggresome location in the transfected cells. Bar = 10 μm. Panels D-F. Three frames from additional file 3, sequence 1 showing the extension of a linear structure from an EB1-ΔN2-GFP aggregate. Panels G-I. Three frames from additional file 3, sequence 2 showing the retraction of this structure. Bar for D-I = 5 μm. Times shown are relative to the first frame in each sequence. Panels A and D-I were obtained using a 63X oil immersion lens. Panels B and C were obtained using a 40X dry lens.

We first examined the behaviour of EB1-ΔN2-GFP using time-lapse fluorescence microscopy in living COS-7 cells. In a minority of cells EB1-ΔN2-GFP displayed a diffuse cytoplasmic distribution with specific labelling of the centrosome (not shown), as described previously in fixed cells [3]. The remaining cells contained single large perinuclear aggregates of the fusion protein against a background of diffuse cytoplasmic fluorescence, with smaller, motile, non-perinuclear structures sometimes apparent (ie see additional file 2). Perinuclear aggregates at this time (14–18 h post-transfection) typically had a compact morphology and were less than half the size of the nucleus in the transfected cell (Fig. 1; additional files 1 and 2).

**Figure 2:**
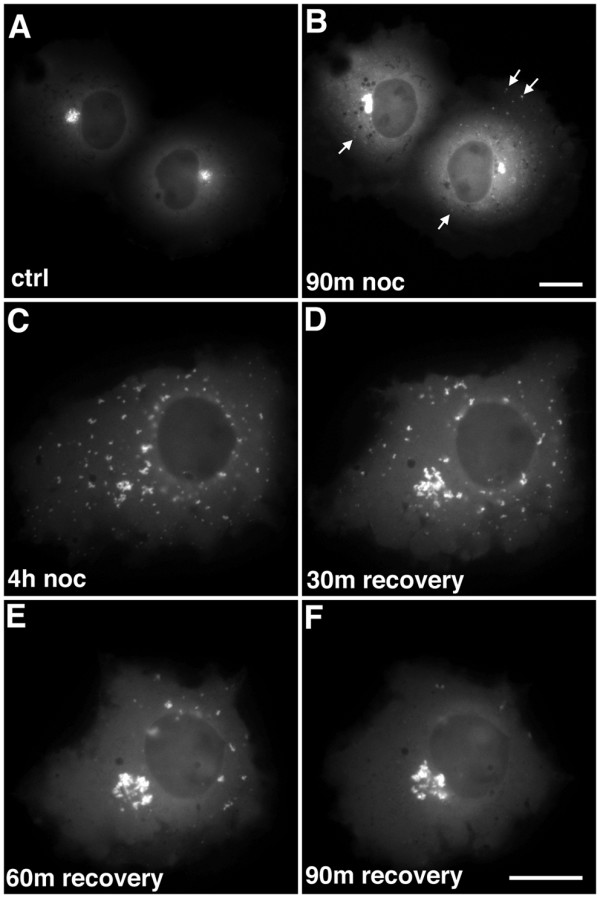
Perinuclear EB1-ΔN2-GFP aggregate formation is microtubule-dependent. Panel A. Still image from living cells containing EB1-ΔN2-GFP aggregates. Panel B. The cells shown in panel A following 90 min incubation with 5 μg/ml nocodazole. Arrows indicate the presence of small peripheral aggregates. Panel C. Still image from a living cell expressing EB1-ΔN2-GFP following 4 h incubation in 5 μg/ml nocodazole. Panels D-F. Images of the cell shown in panel C at 30 min intervals following nocodazole wash out. The formation of a single perinuclear aggregate is seen. Bar = 10 μm.

**Figure 3:**
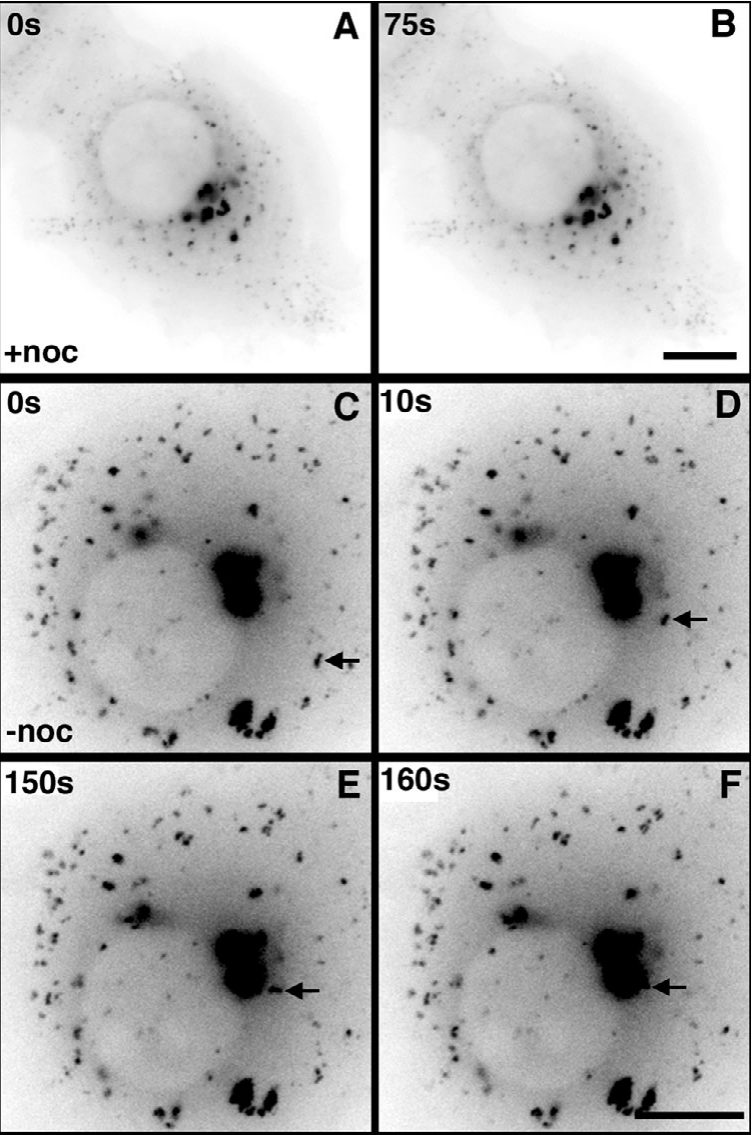
Microtubule-dependent movement of peripheral EB1-ΔN2-GFP aggregates. Panels A and B. Single frames from additional file 4 showing a living cell expressing EB1-ΔN2-GFP after 2 h incubation in nocodazole. All EB1-ΔN2-GFP aggregates are immobile. Bar = 10 μm. Panels C-F. Single frames from additional file 5 showing a living cell expressing EB1-ΔN2-GFP during the recovery phase following nocodazole wash out. The retrograde transport of a peripheral aggregate and its incorporation into a large perinuclear aggregate is arrowed. Times shown are relative to the first frame in the sequence. Bar = 10 μm.

Time-lapse imaging of transfected cells revealed that the EB1-ΔN2-GFP aggregates consisted of variably sized, brightly fluorescent foci closely associated with structures of lower fluorescence intensity (Fig. 1A; additional file 1; additional file 2, sequence 1), with a dense appearance in phase contrast images (Fig. 1B and 1C, arrows; additional file 2, sequence 2). Aside from this, phase contrast imaging did not reveal any obvious differences in cellular morphology when transfected cells were compared with adjacent, untransfected cells (Fig. 1C, additional file 2, sequence 2). EB1-ΔN2-GFP aggregates exhibited constant minor changes in shape while maintaining a relatively cohesive overall structure. However, occasional larger changes in morphology were also observed, typically a rapid extrusion of ribbon-like structures away from the main body of the aggregate (Fig 1. panels D-I, arrows; additional file 3, sequence 1; see also additional file 1). This behaviour suggested that the aggregates consisted of brighter particles linked by a less fluorescently intense matrix. These extrusions appeared relatively stable, but re-incorporation into the main body of the aggregate was also seen (Fig. 1 panels G-I; additional file 3, sequence 2). The speeds of extrusion and retraction were consistent with previous observations of microtubule motor-mediated transport in living cells. For example, in the sequences shown in additional file 3 particle tracking analysis indicated that extrusion occurred at an average speed of 0.14 μm/s whereas retraction occurred at a speed of around 0.1 μm/s, although peak speeds of movement in other time lapse sequences sometimes approached 2.5 μm/s. Addition of the microtubule depolymerising drug nocodazole to the cell culture medium at concentrations known to induce microtubule disassembly in COS-7 cells followed by imaging every 30 min over a time course of 90 min after drug addition revealed the appearance of small spots of fusion protein scattered throughout the cytoplasm and an increase in diffuse cytoplasmic fluorescence. No evidence of shrinkage or dispersal of the perinuclear aggregates was seen over this timescale (Fig. 2 panels A and B, arrows). However, both perinuclear and dispersed aggregates were completely immobile in the presence of nocodazole (Fig. 3, panels A and B; additional file 4). When nocodazole was removed and fresh medium added these aggregates gained motility (Fig. 3, additional file 5) and perinuclear accumulations rapidly assembled, progressively increasing in size and intensity (Fig. 2, panels C-F). Time-lapse imaging revealed smaller structures moving rapidly towards and incorporating into the perinuclear aggregates (Fig. 3; additional file 5). Coalescence of cytoplasmic aggregates before their transport to the perinuclear accumulations was also evident (additional file 5). Particle movement after nocodazole wash out was discontinuous and occasionally bidirectional, but with an obvious bias towards retrograde movement (in both frequency and persistence) as would be expected for the assembly of a perinuclear aggregate. Again, average speeds of particle movement were consistent with microtubule motor-mediated translocation at around 0.1 μm/s. In sum, this data indicated that that the formation and dynamic behaviour of the perinuclear EB1-ΔN2-GFP aggregates was critically dependent upon the presence of an intact microtubule cytoskeleton.

### EB1-ΔN2-GFP aggregates are aggresomes

The behaviour and morphology of the EB1-ΔN2-GFP structures in living cells was reminiscent of the previously reported characteristics of aggresomes [12-14]. Aggresomes form around the centrosome when the accumulation of an aberrantly folded protein exceeds the ability of the cells protein degradation apparatus to dispose of it [18], and they typically share certain common features. For example, like the EB1-ΔN2-GFP aggregates examined here, aggresome formation but not maintenance is microtubule dependent and in the presence of microtubule poisons newly synthesised protein is found in aggregates dispersed throughout the cytoplasm [12]. We therefore fixed and immunostained EB1-ΔN2-GFP transfected COS-7 cells for other aggresomal markers.

**Figure 4:**
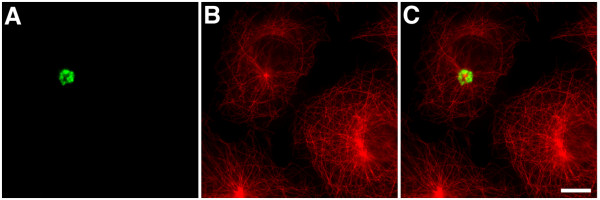
EB1-ΔN2-GFP aggregates are associated with the centrosome. Panels A-C. Cells expressing EB1-ΔN2-GFP were fixed and co-immunostained for GFP (green, panel A) and α-tubulin (red, panel B). A merged image is shown in panel C. EB1-ΔN2-GFP aggregates are clustered around the centrosome. Bar = 10 μm.

**Figure 5:**
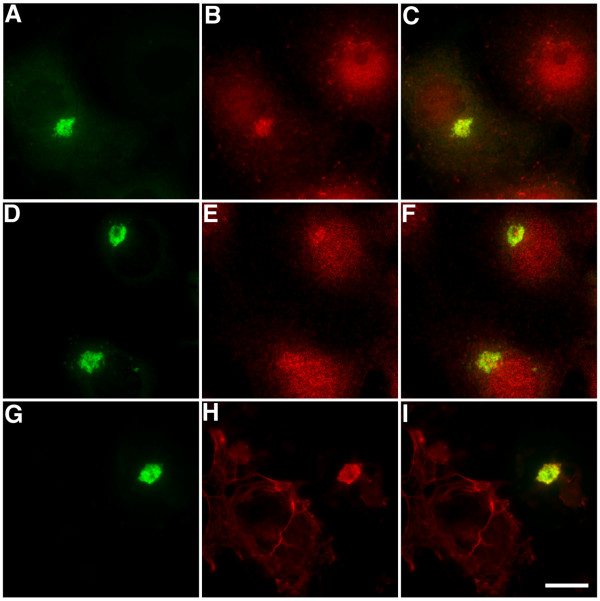
EB1-ΔN2-GFP aggregates are aggresomes. Cells expressing EB1-ΔN2-GFP were fixed and co-immunostained for GFP (green, panels A, D and G) and either the 20s proteosomal subunit (red, panel B), ubiquitin (red, panel E) or vimentin (red, panel H). Merged images are shown in panels C, F and I. All three aggresome markers were present in EB1-ΔN2-GFP aggregates. Bar = 10 μm.

Co-immunostaining for GFP and either α-tubulin (Fig. 4 panels A-C) or γ-tubulin (not shown) confirmed that EB1-ΔN2-GFP aggregates formed around the centrosome in transfected cells. No γ-tubulin immunoreactivity was seen in the aggregates themselves, unlike some aggresomes observed in different systems [19,20]. Co-staining for α-tubulin also indicated that in cells with small aggregates the radial microtubule cytoskeleton was either slightly deformed in the region of the aggregation or unaffected by the presence of the aggregates (Fig. 4 panels A-C). In some cells, particularly those harbouring large aggregates, evidence of extensive microtubule disorganisation was seen (not shown). This is not a widely reported consequence of aggresome formation and in this case may reflect a dominant-negative inhibition of endogenous EB1 function, since we have previously noted the same effect in COS-7 cells expressing different EB1 *N*-terminal deletion mutants [3]. Further co-staining experiments demonstrated that the aggregates were immunopositive for both the 20s subunit of the 26S proteasome (Fig. 5 panels A-C) and ubiquitin (Fig. 5 panels D-F; refs [18,21]), and indicated that they induced the collapse of the vimentin cytoskeleton (Fig. 5 panels G-I; ref [13]). However, the ER resident chaperone BiP/GRP78 was not detected in EB1-ΔN2-GFP aggregates (not shown; ref [18]). These data indicate that the EB1-ΔN2-GFP aggregates bear many of the characteristic hallmarks of an aggresome formed by a misfolded cytosolic protein.

### EB1-ΔN2-GFP aggregates contain APC

**Figure 6:**
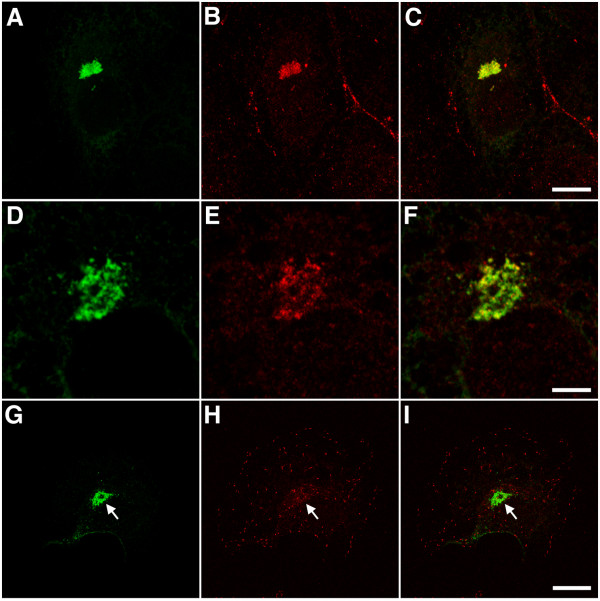
APC is present in EB1-ΔN2-GFP aggresomes. Panels A-C. Cells expressing EB1-ΔN2-GFP were fixed and co-immunostained for GFP (green, panel A) and APC (red, panel B). A merged image is shown in panel C. APC is seen in aggresomes in transfected cells and at peripheral sites in adjacent untransfected cells. Bar = 10 μm. Panels D-F. A single 0.365 μm confocal section through a cell expressing EB1-ΔN2-GFP. APC immunoreactivity (panel E) in aggresomes is heterogeneous but only seen in association with GFP immunoreactivity (panels D and F). Bar = 5 μm. Panels G-I. Cells expressing EB1-ΔN2-GFP were fixed and co-immunostained for GFP (green, panel G) and EB3 (red, panel H). EB3 is seen at microtubule tips but is not present in EB1-ΔN2-GFP aggresomes. Bar = 20 μm. Images in panels A-C and G-I are projections of confocal image stacks.

EB1-ΔN2-GFP retains the ability to interact with APC [3]. To examine whether this interaction might target APC to EB1-ΔN2-GFP aggresomes we co-immunostained transfected cells with antibodies to APC and GFP and examined them using confocal microscopy. Robust APC immunostaining was detected in the EB1-ΔN2-GFP aggregates (Fig. 6 panels A-F). Closer examination of single confocal sections indicated that APC immunostaining in the aggresomes was more heterogeneous that that obtained for GFP, but where present completely overlapped with GFP-positive structures (Fig. 6 panels D-F). This heterogeneity may reflect APC antibody accessibility problems in the centre of the dense aggresomal matrix. We were unable to investigate whether endogenous EB1 was present in the aggresomes as EB1-ΔN2-GFP contains the epitope recognised by the EB1 antibody used here [3]. However, another member of the conserved EB1 protein family, EB3, is also known to interact with APC and p150glued [9] and to localise to growing microtubule tips [22]. COS-7 cells co-express EB1 and EB3 (JMA, unpublished observations). We therefore examined EB3 distribution in transfected cells and found no evidence for EB3 in the EB1-ΔN2-GFP aggregates (Fig. 6 panels G-I). This indicates that EB3 does not associate with EB1-ΔN2-GFP and suggests that it cannot interact with APC or p150glued in the aggresomes, possibly because the EB1/EB3 binding sites in these proteins are already filled by EB1-ΔN2-GFP.

### Dynein/dynactin components are present in EB1-ΔN2-GFP aggresomes

**Figure 7:**
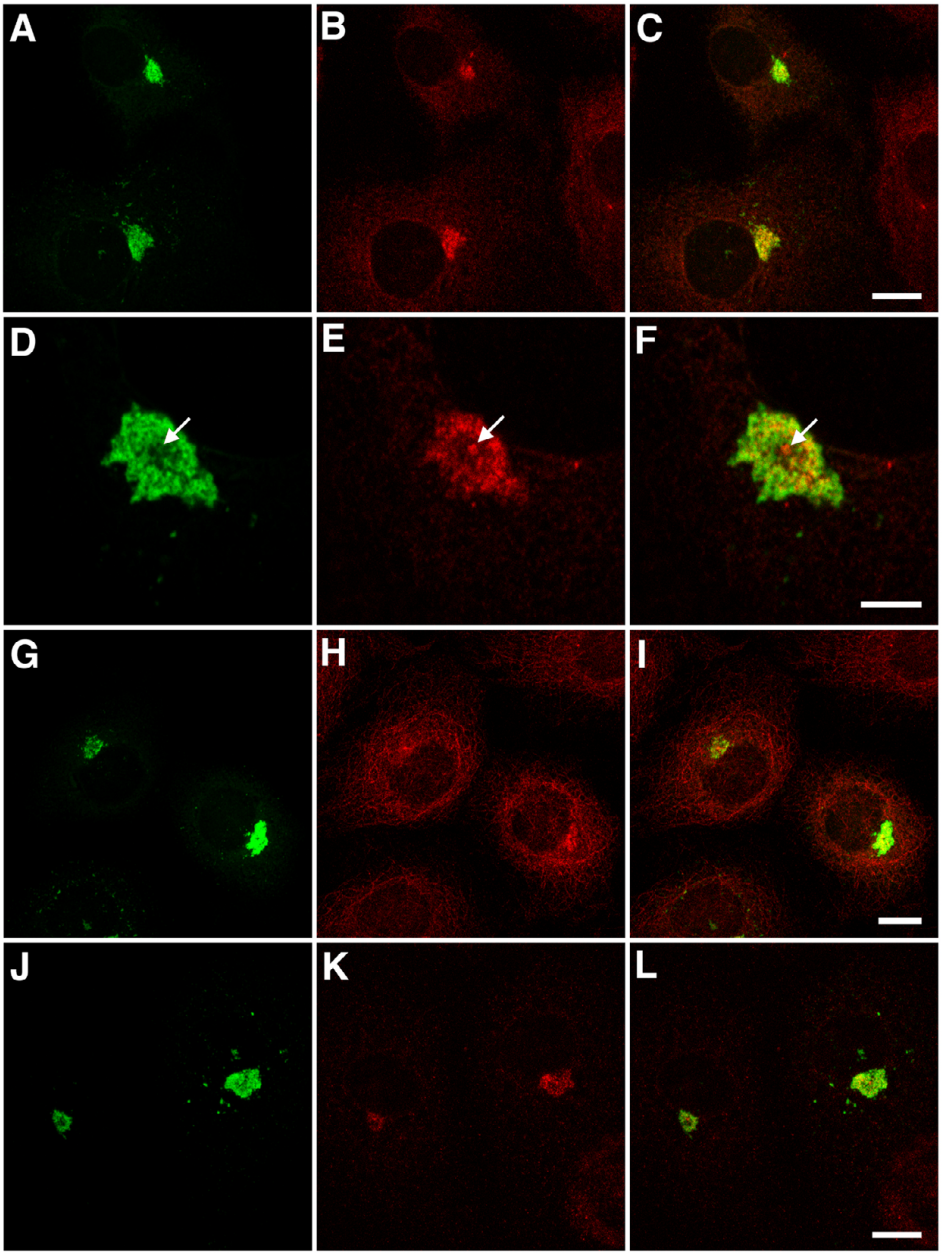
Dynein/dynactin subunits are present in EB1-ΔN2-GFP aggresomes. Panels A-C. Cells expressing EB1-ΔN2-GFP were fixed and co-immunostained for GFP (green, panel A) and p150glued (red, panel B). A merged image is shown in panel C. p150glued is seen in aggresomes. Bar = 10 μm. Panels D-F. A single 0.365 μm confocal section through a cell expressing EB1-ΔN2-GFP. p150glued immunoreactivity in aggresomes (panel E) is heterogeneous but only seen in association with GFP immunoreactivity (panels D and F). Centrosomal immunostaining is also observed (arrow). Bar = 5 μm. Panels G-L. Cells expressing EB1-ΔN2-GFP were fixed and co-immunostained for GFP (green, panel G) and either CDIC (red, panel H) or p50dynamitin (red, panel K). Merged images are shown in panels I and K. Heterogeneous CDIC and p50dynamitin immunostaining is seen in aggresomes and at centrosomes. Bars = 10 μm. Images in panels A-C and G-L are projections of confocal image stacks.

EB1-ΔN2-GFP can also directly interact with the p150glued subunit of dynactin [3]. Co-immunostaining consistently revealed strong p150glued immunostaining in EB1-ΔN2-GFP aggregates (Fig. 7 panels A-F). As with APC, close examination of single confocal sections indicated that p150glued immunoreactivity overlapped with but was more heterogeneous than that seen for GFP in these structures (Fig. 7 panels D-F). Immunostaining for p150glued was also prominent at centrosomes (a normal intracellular localisation for this protein) surrounded by EB1-ΔN2-GFP aggregates (Fig. 7 panels D-F, arrows). Co-immunostaining also revealed the presence of both the CDIC and p50dynamitin subunits of dynein/dynactin in EB1-ΔN2-GFP aggresomes (Fig. 7 panels G-L). As with p150glued both of these proteins displayed a heterogeneous distribution in the aggresome and were also present at the centrosome. In general, and bearing in mind the problems involved in trying to compare staining intensities for different proteins, aggresomal immunostaining for these proteins appeared to be weaker and more variable than that obtained for p150glued. For example, strong immunostaining for both CDIC and p50dynamitin was observed most consistently in larger aggregates but was less apparent in smaller aggregates.

### Fractionation and Western blotting analysis of EB1-ΔN2-GFP expressing cells

**Figure 8:**
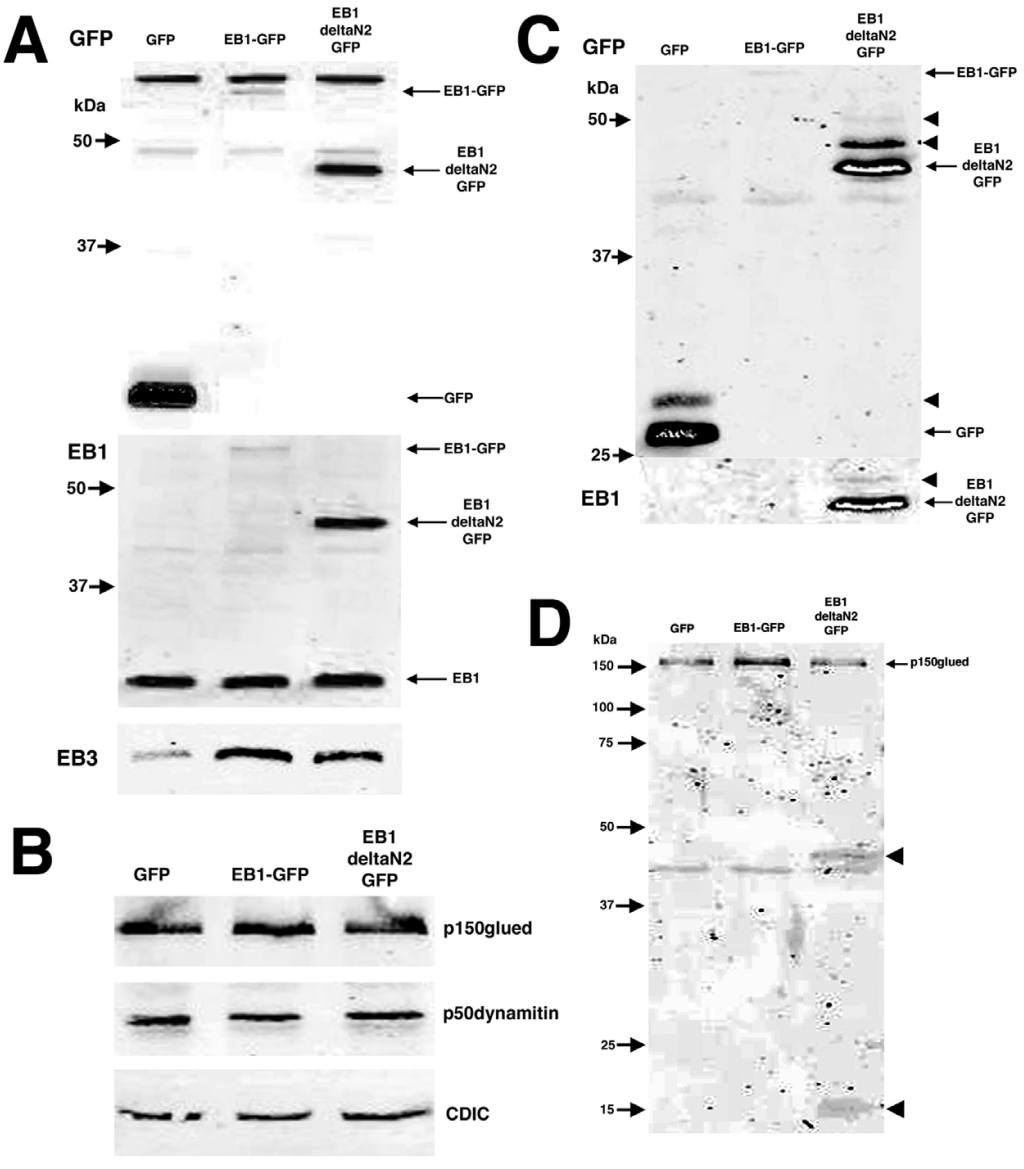
Fractionation and Western blotting analysis of EB1-ΔN2-GFP aggregates. COS-7 cells were transfected with GFP, EB1-GFP or EB1-ΔN2-GFP for 24 h before extraction in detergent-containing buffer and separation into soluble (panels A and B) and insoluble (panels C and D) fractions and analysis by SDS-PAGE and Western blotting. **Panel A. **GFP immunoblotting of soluble fractions revealed the presence of single immunoreactive bands for GFP, EB1-GFP and EB1-ΔN2-GFP (arrows). Non-specific cross-reacting bands are evident using this polyclonal antibody. EB1 immunoblotting revealed single bands for EB1-GFP, EB1-ΔN2-GFP and endogenous EB1 (arrows). EB3 immunoblotting revealed a single immunoreactive band in all soluble fractions. **Panel B. **Immunoblotting for p150glued, p50dynamitin and CDIC indicated that the level of these proteins in soluble fractions were similar in all transfections. **Panel C. **GFP immunoblotting revealed the presence of two immunoreactive bands in the insoluble fraction of GFP transfected cells (arrow and arrowhead), at least three bands in EB1-ΔN2-GFP transfected cells (arrow and arrowheads) and a single weak band for EB1-GFP (arrow). An overexposed image is shown to highlight the accessory bands. EB1 immunoblotting revealed the presence of two immunoreactive bands in EB1-ΔN2-GFP insoluble fractions (arrow and arrowhead). This blot is also shown overexposed. **Panel D. **Immunoblotting detected p150glued in the insoluble fraction of all extracts (arrow). Two lower molecular weight bands were specifically detected in the insoluble fraction of EB1-ΔN2-GFP transfected cells (arrowheads). The 43 kDa non-specific band present in the GFP and p150glued panels was present in immunoblots performed on insoluble cellular fractions regardless of the primary antibody used.

A characteristic of aggresomes is their insolubility in non-ionic detergent solutions [13,14]. We therefore performed a simple fractionation by extracting cells in 0.1% Triton X-100 in PBS and separating soluble and insoluble fractions by centrifugation. The resulting fractions were examined using SDS-PAGE and Western blotting. As shown in Fig. 8A, immunoblotting with a GFP-specific antibody revealed the presence of single bands representing GFP, EB1-GFP or EB1-ΔN2-GFP in the soluble fraction of the relevant transfected cell extract (arrowed). Immunoblotting with an EB1-specific antibody gave a similar result for EB1-GFP and EB1-ΔN2-GFP and also revealed that the levels of endogenous soluble EB1 were unchanged by transfection. Immunoblotting with an EB3-specific antibody detected a single immunoreactive band. We also probed soluble cell fractions with antibodies specific for the p150glued, p50dynamitin and CDIC subunits of the dynein/dynactin complex. This indicated that the levels of these proteins in the soluble fraction were unaffected by the expression of GFP or either of the EB1-derived fusion proteins (Fig. 8B).

These analyses were then repeated on the detergent-insoluble fraction from transfected cells. GFP immunoblotting revealed two immunoreactive bands in the insoluble fraction of cells expressing GFP (Fig. 8C). The smaller of these (arrowed) corresponded to the expected molecular weight of GFP whereas the larger (arrowhead) is consistent with the size of a monoubiquitylated GFP molecule, suggesting that a proportion of overexpressed GFP in COS-7 cells might be turned over by normal cellular mechanisms of protein degradation. Only weak immunolabelling was detected for EB1-GFP in overexposed blots (Fig. 8C, arrowed), consistent with previous observations showing endogenous EB1 to be highly soluble (1). In contrast, EB1-ΔN2-GFP was highly insoluble in transfected cell extracts (Fig. 8C). Furthermore, in addition to a major band corresponding to the expected size of EB1-ΔN2-GFP (arrowed), at least two higher molecular weight bands were also detected (arrowheads). The equal spacing between these bands suggests that these represent ubiquitylated forms of the insoluble protein, consistent with the positive ubiquitin immunoreactivity observed in EB1-ΔN2-GFP aggregates by immunostaining of transfected cells (Fig. 5 panels D-F). The presence of at least one higher molecular weight species of EB1-ΔN2-GFP was confirmed when blots were probed with the EB1 antibody and examined after overexposure (Fig. 8D, arrowhead).

EB3 and the dynein/dynactin components p50dynamitin and CDIC were essentially undetectable in the insoluble fraction of any of the transfected cell extracts (not shown). p150glued was detected in the insoluble fraction of all extracts (Fig. 8C, arrowed) but no evidence for an enrichment of insoluble p150glued in cells expressing EB1-ΔN2-GFP was found. However, two lower molecular weight species of approximately 45 and 15 kDa were specifically detected by the p150glued monoclonal antibody in the insoluble fraction of cells expressing EB1-ΔN2-GFP (Fig. 8C, arrowheads).

## Discussion

The data presented in this study indicate that the perinuclear aggregates formed in cells expressing EB1-ΔN2-GFP are aggresomes, although the underlying reason for their formation by this fusion protein remains unclear. Two mechanisms seem possible. First, the initial aggregation occurs as a direct consequence of the interaction between EB1-ΔN2-GFP and p150glued, perhaps coupled to aberrant retrograde transport of this complex, and aggresome formation occurs subsequent to this. A second possibility is that EB1-ΔN2-GFP misfolds, becomes refractive to proteolytic degradation and thereby triggers the formation of an aggresome. Neither EB1-GFP nor any of the other GFP-tagged EB1 deletion mutants generated in our laboratory, including those that do not interact with microtubules but retain an ability to bind APC and p150glued, form aggresomes in transfected cells [3]. This suggests that the first possibility is less likely. Furthermore, our Western blotting analyses indicated that cell extracts from cultures transfected with the EB1-ΔN2-GFP construct contained significantly more fusion protein than those from cultures transfected with the EB1-GFP construct (Fig. 8). Since the transfection efficiency for the EB1-GFP and EB1-ΔN2-GFP constructs was similar, equal amounts of transfected cell extracts were analysed and the base expression vector used for both constructs was identical, the best explanation for the higher levels of EB1-ΔN2-GFP relative to EB1-GFP in our experiments is that EB1-GFP was turned over normally within the cell whereas EB1-ΔN2-GFP was not. This data therefore supports the second mechanism suggested above. However, if aggregation is a response to EB1-ΔN2-GFP misfolding, then the presence of both p150glued and APC in the aggregates suggests that this misfolding is partial since the binding sites for both of these proteins appears to be functional (Figs. 6 and 7; ref [3]). We therefore propose that EB1-ΔN2-GFP in COS-7 cells adopts a conformation where APC and p150glued binding are maintained but the fusion protein is recognised by the cellular stress response pathway for misfolded proteins, perhaps as a result of a disordered *N*-terminal region arising from the loss of the CH domain from the EB1 *N*-terminus. As this partially misfolded protein is resistant to degradation it accumulates within the cytoplasm and an aggresome is formed.

Other investigators have previously described the formation of aggresomes by a cytosolic GFP fusion protein (GFP-250) in living cells [14]. EB1-ΔN2-GFP aggresome formation appeared to correlate well with that observed for GFP-250, particularly during aggresome assembly following nocodazole treatment and wash out. However, previous ultrastructural studies have suggested that aggresomes represent an "aggregate of aggregates" formed by an accumulation of individual particles without further coalescence into a cohesive structure [12-14]. Our time-lapse studies suggest that this is not the case with EB1-ΔN2-GFP. In our system aggresomes exhibited dynamic microtubule-dependent linear extensions. These typically resembled beads on a string with one end remaining attached to the main structure of the aggresome. Detachment and anterograde movement of individual particles from aggresomes was never seen. These observations indicate that the brighter structures in EB1-ΔN2-GFP aggresomes must be linked in some way and, since extensions were never seen to fully detach from aggresomes, this linkage is strong enough to resist the forces generated by microtubule motors. Together with our data showing that microtubules are not necessary to maintain the pericentrosomal location of pre-existing EB1-ΔN2-GFP aggresomes, this suggests that these structures possess an inherent cohesiveness and are not simply a collection of individual elements maintained in close proximity to the centrosome by dynein motor activity. At present it remains unclear whether this dynamic behaviour is a universal feature of aggresomes or is restricted to those formed by EB1-ΔN2-GFP. However, we note that associations with kinesin II have recently been described for both p150glued [23] and APC [24]. Since both proteins are clearly present in EB1-ΔN2-GFP aggregates these interactions could potentially contribute to the dynamic behaviour of these structures.

Recent studies of aggresome formation by mutant CFTR and SOD proteins in cells treated with proteosome inhibitors indicated that the dynein/dynactin components CDIC, p50dynamitin and p150glued were all recruited to aggresomes [15,25]. This raises the possibility that the p150glued observed in EB1-ΔN2-GFP-induced aggresomes was present as a normal cofactor for aggresome formation rather than as a specific EB1-ΔN2-GFP ligand. Furthermore, an increase in the amounts of p150glued and p50dynamitin in the detergent insoluble fraction of cells containing mutant SOD-induced aggresomes was reported [25]. In our experiments no increase in the amount of insoluble full-length forms of these proteins was observed. However, in contrast to previous studies we found lower molecular weight p150glued species in the detergent-insoluble fraction of cells expressing EB1-ΔN2-GFP. The p150glued antibody used in this study recognises an epitope at the *N*-terminus of the protein [26], the same region that mediates the EB1-p150glued interaction [3] but distinct from the regions responsible for the interactions with other dynein/dynactin subunits [8]. It seems possible that the bands detected in our immunoblots represent *N*-terminal p150glued fragments tightly bound to insoluble EB1-ΔN2-GFP and inaccessible to the proteosome. An analogous mechanism has been proposed to explain the limited proteolytic processing of NF-κB precursors, where it is suggested that a close association with a partner molecule inhibits the processive degradation of the NF-κB p50 domain [27]. To explain the discrepancies between our data and that presented in the study of mutant SOD-induced aggresomes we therefore propose that in the latter case the dynactin complex is recruited to but not trapped within aggresomes as part of a normal cellular response, whereas in our system the aggresome-associated p150glued is tightly bound to EB1-ΔN2-GFP. Subsequent degradation of exposed regions of the molecule destroys the binding sites for p150glued-associated dynein/dynactin subunits, precluding their stable co-incorporation into the aggresome. However, binding to proteolytically-resistant EB1-ΔN2-GFP molecules preserves the p150glued fragments detected by the monoclonal antibody used in our study.

## Conclusion

In this work we were fortunate in being able to examine aggresome formation by a defined cytosolic protein that possesses direct interactions with two other well-characterised proteins, p150glued and APC. This allowed us to show that these proteins were present in the aggresomes arising from EB1-ΔN2-GFP expression in transfected cells. Our data therefore suggests that a misfolded protein can trap its normal endogenous cellular ligands in an aggresome, potentially resulting in the degradation of these partners. This could have implications for our understanding of human diseases thought to involve protein misfolding and the formation of aggresome-like structures, such as Parkinsons disease and other neurodegenerative disorders [12]. Our data provides a proof of principle that the pathologies characteristic of these diseases could arise in part from the inappropriate trapping and degradation of the normal physiological partners of misfolded proteins in aggresomes and inclusion bodies.

## Methods

### Cells

COS-7 cells were cultured and drug treatments performed as described previously [1]. Transfections were performed using GeneJuice (Novagen) according to the manufacturers instructions. Manual counting of GFP immunostained transfected cell populations indicated that transfection efficiencies at 18 h post-transfection were consistently in the region of 60% for the GFP expression plasmid and somewhat lower at around 25% for the EB1-GFP and EB1-ΔN2-GFP expression plasmids.

### Antibodies and reagents

Monoclonal antibodies specific for EB1, EB3, p150glued and p50dynamitin were obtained from Transduction Laboratories. Monoclonal antibodies specific for γ-tubulin and cytoplasmic dynein intermediate chain (CDIC) were obtained from Sigma. A mouse monoclonal antibody specific for BiP was obtained from BD Biosciences. A rat anti α-tubulin antibody was obtained from Serotec. Rabbit polyclonal antibodies against ubiquitin and the 20S proteosomal subunit were obtained from DAKO and Affiniti Research Products respectively. Rabbit polyclonal and mouse monoclonal anti-GFP antibodies were obtained from Clontech. A rabbit polyclonal antibody specific for APC (M-APC; ref [16]) was a kind gift from Dr Inke Nathke, University of Dundee, UK. All secondary antibodies were highly cross-adsorbed Alexa 488 and 594 conjugates obtained from Molecular Probes. Nocodazole was obtained from Sigma.

### Plasmids

The GFP, EB1-GFP and EB1-ΔN2-GFP expression plasmids used in this work have been described previously [3,7,17].

### Immunofluorescence

COS-7 cells were cultured and transfected on glass coverslips. Cultures were processed for immunocytochemistry using methanol fixation 18 h after transfection and imaged using a Leica TCS-SP confocal microscope as described previously [7]. Alternatively, cells were imaged by fluorescence microscopy using a Zeiss Axiovert 200 inverted microscope coupled to an Orca II ER CCD camera controlled by AQM6 software (Kinetic Imaging, Nottingham, UK). Figures were assembled using Adobe Photoshop 7.

### Time-lapse fluorescence imaging

Cells were grown and transfected in 35 mm glass-bottomed culture dishes (Iwaki brand; Asahi Techno Glass Corporation, Japan) obtained from Bibby Sterilin. 14–18 h after transfection the cell culture medium was replaced by 1.5 ml of pre-warmed medium containing 20 mM HEPES. The cells were then transferred to a Zeiss Axiovert 200 inverted microscope with the stage enclosed in a heated chamber (Solent Scientific, UK) maintained at 37°C. After 15 min equilibration cells were examined by fluorescence microscopy using a Zeiss Plan Apochromat 63X/1.4NA oil immersion lens or by fluorescence and phase contrast microscopy using a Zeiss A-Plan 40X/0.65NA dry lens. An excitation/emission filterset optimised for eGFP imaging was used for fluorescence microscopy (Chroma Technology Corp., Brattleboro, USA; filterset ID 86007). Time-lapse images were obtained using Ludl shutters and a Hamamatsu Orca II ER camera. Microscope, camera, filterwheels and shutters were controlled by AQM 6 software (Kinetic Imaging, Nottingham, UK). Typically, images were obtained using 1 × 1 binning and exposure times of less than 250 ms/frame with time-lapse intervals ranging between 5s and 30s. Time-lapse image series were saved as uncompressed AVI files then cropped, compressed and converted into Quicktime movies using Adobe ImageReady 7. Some movies are presented using an inverted greyscale colour look-up table to enhance the visibility of small structures. Particle tracking analyses were performed using Motion Analysis software from Kinetic Imaging.

### Cell extractions, SDS-PAGE and Western blotting

Cells were harvested by scraping and collected by centrifugation 18 h after transfection. Cell pellets were resuspended in ice-cold PBS containing 0.1% Triton X-100, a mixture of protease inhibitors (Complete EDTA-free tablets, Roche Diagnostics, Germany), 2 mM EDTA, 50 mM sodium fluoride and 100 μM sodium orthovanadate (PBS/TX100) then incubated on ice for 5 min with occasional mixing. Insoluble and soluble fractions were separated by centrifugation at 12000 g for 5 min in a benchtop microcentrifuge. Once the supernatant was removed to a fresh tube the pellet was resuspended in a volume of PBS/TX100 equivalent to that of the removed supernatant. An equal volume of 2x concentrated SDS-PAGE sample loading buffer containing 5 mM DTT was added to both the soluble and insoluble fractions, followed by boiling for 5 min. To reduce viscosity pellet samples were passed repeatedly through a narrow gauge needle attached to a syringe before gel loading. Unused sample was snap frozen in liquid nitrogen and stored at -80°C until needed. SDS-PAGE and Western blotting were performed essentially as described previously [1]. Blots were probed using fluorescently conjugated secondary antibodies and visualised using a Li-Cor Odyssey quantitative Western Blotting system. Equal loading of different cell extracts onto SDS-PAGE gels and subsequent transfer on to Western blotting membranes was confirmed by quantitative immunoblotting with a monoclonal β-actin antibody. Gel images were processed and converted into greyscale images using Adobe Photoshop 7. The images of some blots shown in this study have been cropped due to size considerations; only regions where no specific immunoreactivity was observed after overexposure have been removed.

## Authors' contributions

NPR contributed to the live cell imaging, immunostaining and Western blotting studies and helped draft the manuscript. KM contributed to the live cell imaging, Western blotting and immunostaining studies and helped to draft and revise the manuscript. TL maintained cell cultures, participated in the Western blotting studies and helped revise the manuscript. MA contributed to the live cell imaging and immunostaining studies and helped to revise the manuscript. JMA and EEM conceived the study, participated in its design and execution, contributed reagents and helped to both draft and revise the manuscript. All authors read and approved the final manuscript.

## Supplementary Material

Additional File 2 EB1-ΔN2-GFP aggregates in living COS-7 cells. Sequence 1: image capture rate was 1 frame/5s using a 40X lens, playback rate is 20 frames/s. The dynamic behaviour of EB1-ΔN2-GFP aggregates is again evident. Sequence 2: image capture rate was 1 frame/5s using a 40X lens, playback rate is 20 frames/s. This movie shows the cells in sequence 1 imaged by phase-contrast microscopy and allows the behaviour of these cells to be compared with two adjacent untransfected cells. The sequence begins 30s after the end of sequence 1. EB1-ΔN2-GFP aggregates are visible as phase-dense structures but their presence has no obvious effect upon cell behaviour. Click here for file

Additional File 1 EB1-ΔN2-GFP aggregate morphology and dynamics. Image capture rate was 1 frame/5s using a 63X oil immersion lens, playback rate is 20 frames/s. Aggregates consist of brighter puncta in a matrix of lower fluorescence intensity. The dynamic nature of the aggregate can be seen. Structures can be observed extending away from the aggregate while remaining attached to it; on obvious example of this can be seen around the nuclear periphery at the bottom left of the aggregate. Click here for file

Additional File 3 Extrusion and retraction of a linear structure from an EB1-ΔN2-GFP aggregate. Image capture rate was 1 frame/5s using a 63X oil immersion lens, playback rate is 20 frames/s. Sequence 1: the extension of a linear fluorescent structure from an EB1-ΔN2-GFP aggregate is shown; the aggregate itself is overexposed to allow better resolution of this structure, which consists of brighter puncta linked to each other and the aggregate by a ribbon of less fluorescent material. Sequence 2: imaging was initiated approximately 1 min after the end of sequence 1. Retraction of the structure extended by the EB1-ΔN2-GFP aggregate can be seen. Click here for file

Additional File 4 Movement of EB1-ΔN2-GFP aggregates is microtubule-dependent. Image capture rate was 1 frame/5s using a 40X lens, playback rate is 20 frames/s. This movie shows aggregate behaviour in cells after 2 h incubation in nocodazole. In the presence of the drug the aggregates are completely immobile. Click here for file

Additional File 5 Perinuclear EB1-ΔN2-GFP aggregate assembly is microtubule-dependent. Image capture rate was 1 frame/10s using a 63X oil immersion lens, playback rate is 20 frames/s. Cells were incubated in nocodazole for 4 h followed by a wash and the addition of fresh imaging medium without drug. Imaging was initiated 30 min after nocodazole wash out. In the absence of drug EB1-ΔN2-GFP aggregates regain motility. Movement is intermittent and with an overall retrograde bias leading to the growth of perinuclear aggregates. Click here for file
